# Neuroinflammation in Radiation Maculopathy: A Pathophysiologic and Imaging Perspective

**DOI:** 10.3390/cancers17152528

**Published:** 2025-07-31

**Authors:** Giulia Midena, Raffaele Parrozzani, Marisa Bruno, Elisabetta Pilotto, Edoardo Midena

**Affiliations:** 1 IRCCS–Fondazione Bietti, 00198 Rome, Italy; giulia.midena@fondazionebietti.it (G.M.); marisa.bruno@fondazionebietti.it (M.B.); 2 Department of Ophthalmology, University of Padova, 35128 Padova, Italy; raffaele.parrozzani@unipd.it (R.P.); elisabetta.pilotto@unipd.it (E.P.)

**Keywords:** radiation maculopathy, neuroinflammation, chorioretinal imaging, hyperreflective retinal foci

## Abstract

Background: Radiation maculopathy (RM) is a delayed, sight-threatening complication of ocular radiotherapy. Traditionally regarded as a pure microvascular disease, emerging evidence points to the central role played by retinal neuroinflammation, driven by microglial activation and cytokine dysregulation affecting both the retina and the choroid. Hyperreflective retinal foci, neuroinflammatory in origin (I-HRF), visualized through advanced imaging modalities such as spectral domain optical coherence tomography (OCT), have been identified as early and critical biomarkers of both preclinical and clinical retinal neuroinflammation. Materials and Methods: This review synthesizes findings from experimental and clinical studies to explore the pathophysiology of neuroinflammation and the associated imaging parameters in RM. Results: The integration of experimental and clinical evidence specifically underscores the significance of I-HRF as an early indicator of neuroinflammation in RM. OCT enables the identification and quantification of these biomarkers, which are linked to microglial activation and cytokine dysregulation. Conclusions: The pathophysiology of RM has evolved from a predominantly vascular condition to one strongly secondary to neuroinflammatory mechanisms involving the retina and choroid. In particular, I-HRF, as early biomarkers, offers the potential for preclinical diagnosis and therapeutic intervention, paving the way for improved management of this sight-threatening complication.

## Simple Summary

Radiation maculopathy is a secondary maculopathy caused by irradiation of the eye for intraocular malignancies. This condition threatens vision, up to legal blindness. Previously considered a pure vascular disease, new evidence documents that radiation-induced retinal and choroidal neuroinflammation, driven by immune cells (microglia) and abnormal protein signals (cytokines), plays a crucial role. Specific intraretinal spots, called (inflammatory) hyperreflective retinal foci, can be seen by means of optical coherence tomography, and they are currently considered signs of neuroinflammation of the retina. Recognizing these early changes helps to understand how radiation maculopathy develops and progresses and offers the possibility for earlier diagnosis and more specific treatment.

## 1. Introduction

Radiation maculopathy (RM) is a sight-threatening complication mainly associated with brachytherapy or proton therapy for uveal melanoma (UM) [1,2,3,4,5,6,7]. It encompasses the specific macular involvement secondary to eye irradiation [8,9,10,11,12]. RM represents one of the most frequent and visually disabling complications following successful radiotherapeutic management of UM. Its relevance, from both a diagnostic and therapeutic point of view, depends on the peculiar role of the macula (the central part of the retina, 5.50 mm in diameter) as the most critical retinal region for visual function. The macula’s high metabolic demand and its dependence on a fine-tuned neurovascular coupling make it particularly susceptible to radiation-induced damage [8,9,10,11,12]. It is characterized by delayed-onset damage to the retinal vasculature and neuronal and glial components (Müller cells, astrocytes, and microglial cells) of the macula, typically manifesting months to years after exposure to ionizing radiation [1,9,13]. Initially, RM was primarily attributed to vascular changes, including capillary occlusion, leakage, and subsequent ischemia [1,2,14]. This model, which dominated the understanding of RM for decades, was based on clinical findings resembling those observed in diabetic or hypertensive retinopathy. However, such a view appears increasingly insufficient to explain the early alterations detectable through modern imaging techniques and the subclinical progression observed in some patients. Recent studies have revealed that neuroinflammatory mechanisms—since now the terms retinal inflammation and neuroinflammation will be used as synonymous, pointing to the specific cellular environment of the retina—play a pivotal role in its pathogenesis [15,16,17,18], and that retinal inflammation contributes to the microvascular damage occurring in RM. The interaction between microvascular pathology and glial cell activation is now considered central in the onset and progression of RM. The activation of microglia, along with the release of pro-inflammatory cytokines, has been identified as a key driver of RM [15,16,19]. This neuroinflammatory response disrupts neurovascular interactions, further exacerbating functional impairment and disease progression. Advanced imaging biomarkers, such as hyperreflective retinal foci (neuroinflammatory HRF: I-HRF) [20], now provide a promising tool for early detection of RM, enabling intervention during the subclinical phase before irreversible retinal damage occurs [15,16]. In addition to I-HRF, the presence of subretinal fluid is another biomarker strictly associated with neuroinflammation [3,21,22]. These imaging findings precede any observable functional deterioration and highlight the need for vigilant retinal monitoring after radiotherapy [20]. Finally, retinal and choroidal ischemia trigger a sustained neuroinflammatory response, contributing to progressive neurodegeneration [3,23,24]. These emerging insights underscore a paradigm shift in understanding RM, moving beyond a purely vascular focus to encompass its neuroinflammatory and neurodegenerative dimensions. This integrated model opens new therapeutic perspectives targeting inflammation, microglial activity, and gliosis, not only vascular repair or anti-VEGF therapy. Moreover, recent data suggest that combining imaging with functional testing may enhance early detection and guide personalized interventions [25]. In this context, RM emerges as a complex, multifactorial retinal disease in which radiotherapy acts as the initial trigger of a long-lasting inflammatory cascade.

### 1.1. Radiotherapy for Uveal Melanoma

Radiotherapy remains the gold standard treatment for UM [4]. While enucleation is sometimes necessary for large tumors, medium- and small-sized UMs are primarily managed with ocular irradiation [5,26]. The most common radiotherapy techniques include plaque brachytherapy and proton therapy, both of which effectively treat the majority of UM cases.

**Figure 1:**
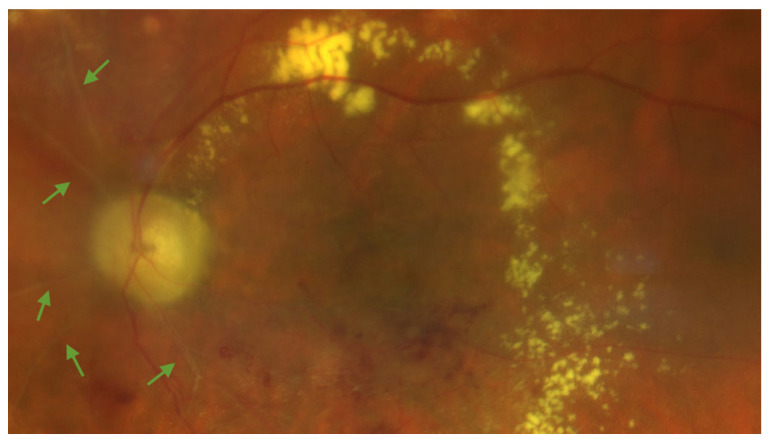
Color fundus photography of the left eye of a patient showing radiation maculopathy, characterized by ghost vessels (green arrows), hard exudates, and retinal hemorrhages. The subject was treated for a large-sized choroidal melanoma located in the inferotemporal quadrant. Plaque brachytherapy using Iodine-125 was performed, delivering a total dose of 85 Gy to the tumor apex. The image was acquired 25 months after radiation treatment. The patient had no history of diabetes mellitus.

Ocular irradiation achieves tumor control but also results in iatrogenic effects on surrounding healthy tissues, particularly the retina, including the macular area [1,2,13]. Inflammation plays a crucial role in radiation-induced retinal damage, contributing to endothelial dysfunction and subsequent vascular impairment [1,2,13]. Radiation exposure triggers an inflammatory response that promotes endothelial cell injury, oxidative stress, and the release of pro-inflammatory cytokines. This inflammatory cascade disrupts the normal homeostasis of the retinal microenvironment, leading to increased vascular permeability, edema, and exudate accumulation. As the endothelial damage progresses, structural alterations in the retinal blood vessels become evident, including outpouchings, telangiectasia, and microaneurysms [1,3]. These anatomic vascular changes result in progressive vessel obliteration, leading to capillary narrowing or closure. The ensuing microvascular occlusion mirrors the effects observed in brain capillaries following radiation exposure. Ultimately, impaired blood flow induces retinal ischemia, which, if unresolved, can progress to neovascularization and to retinal atrophy [1,3]. Figure 1 represents the clinical appearance of RM.

Moreover, recent studies suggest that cellular stress induced by radiation leads to DNA damage in tumor cells, promoting cellular senescence [27]. This state of senescence is a key mechanism driving tumor regression after radiotherapy. However, senescent cells remain metabolically active for prolonged periods, adopting a senescence-associated secretory phenotype (SASP) [27,28]. SASP promotes interactions between senescent cells and the surrounding microenvironment, promoting a localized neuroinflammatory response involving retinal microglial cells and affecting nearby healthy vascular cells, contributing to further tissue damage [27].

### 1.2. Non-Invasive Imaging Techniques

Multimodal retinal and choroidal imaging has become an indispensable tool for the diagnosis and management of RM, offering detailed insights into macular changes caused by ocular irradiation [24,29,30,31]. In particular, non-invasive methods are gaining increasing importance, and high-resolution imaging modalities are particularly valuable for detecting early signs of RM, monitoring disease progression, and assessing the efficacy of treatments [3,21,31,32].

Optical coherence tomography (OCT) is one of the most widely used imaging techniques for evaluating RM [12,33,34,35]. RM, through the OCT analysis, is characterized by edematous changes in its early stages (cystic macular edema) and/or by ischemia and atrophic changes in the macular area in more advanced stages [12,30]. Indeed, OCT provides cross-sectional views of the macula, allowing for the identification of hallmark features such as intraretinal and subretinal fluid, inflammatory hyperreflective retinal foci (I-HRF), and disruptions of the external limiting membrane and ellipsoid zone [36,37]. Some of these findings often appear before clinical symptoms, highlighting OCT’s importance in early detection [16]. Advances such as spectral domain OCT and swept-source OCT offer enhanced resolution and deeper visualization of macular structures [34,36]. OCT angiography (OCTA) further complements OCT by enabling non-invasive visualization of the retinal and choroidal vasculature [38,39]. It identifies microvascular abnormalities, including capillary dropout, microaneurysms, and telangiectasia, which are characteristic of RM [29,32,40]. Moreover, OCTA enables the visualization of the choroid by penetrating all chorioretinal layers, providing depth-resolved, three-dimensional imaging of the choroidal vasculature [38]. Unlike fluorescein angiography, OCTA provides depth-resolved imaging without requiring dye injection, making it a safer and more comfortable option for patients [41].

Finally, recent advancements, including artificial intelligence-enhanced image analysis, are paving the way for more precise and earlier detection of RM. These emerging technologies hold promise for improving diagnostic accuracy and optimizing treatment strategies to preserve visual function [37].

## 2. Materials and Methods

To identify relevant articles in the medical literature, a search was conducted in MEDLINE^®^ (8600 Rockville Pike, Bethesda, MD, USA) for English-language publications from January 2000 to April 2025. The search utilized specific terms both individually and in combination to refine results: “retina irradiation”, “radiation maculopathy,” “retinal inflammation”, “retinal neuroinflammation”, “optical coherence tomography,” “optical coherence tomography angiography”, “hyperreflective retinal foci”, “subretinal fluid”, “retinal ischemia”, “choroidal ischemia”. Additional articles were identified by examining the reference lists of retrieved publications. Two investigators independently reviewed all identified articles, selecting the most relevant studies for inclusion based on their significance. Papers without baseline imaging data were excluded. Preference was given to review articles and studies over case reports or case series. All included articles were thoroughly reviewed by the authors to ensure their relevance and contribution to the topic.

## 3. Results

A total of 182 records were initially identified through the MEDLINE^®^ database and reference screening. After title and abstract screening, 103 records were excluded due to irrelevance to the topic, language restrictions, or publication type. Full-text review was conducted on 79 articles, of which 48 met the inclusion criteria and were included in the final analysis. Inclusion criteria comprised original studies in English, including both human and animal models, focusing on radiation-induced retinal damage, neuroinflammation, and imaging biomarkers in radiation maculopathy. Exclusion criteria were the absence of baseline imaging data, non-original content, or lack of focus on retinal neuroinflammation. All articles were independently reviewed by two investigators (G.M. and R.P.), and discrepancies were resolved through discussion.

### 3.1. Pathophysiology of Retinal Neuroinflammation in Radiation Maculopathy

#### 3.1.1. Neurovascular Interactions

The neurovascular unit, comprising retinal neurons, glial cells, and vascular structures, plays a crucial role in maintaining retinal function [1]. Ionizing radiation induces significant damage to this unit by triggering an inflammatory response that directly affects pericytes and endothelial cells, both essential for vascular stability and integrity. As vascular integrity deteriorates, ischemia and hypoxia develop in the retinal tissue [1,9]. Hypoxia further amplifies inflammation by activating hypoxia-inducible factors, which stimulate additional inflammatory pathways and exacerbate neurovascular damage [1,9]. This chronic inflammatory state perpetuates vascular instability and neuronal injury, ultimately compromising retinal function. The interplay between vascular compromise and neuroinflammation creates a vicious cycle wherein ischemic conditions drive inflammation, and inflammation exacerbates vascular instability [1,9,15,19]. This dynamic process underscores the multifactorial nature of RM and highlights the need for strategies that address both vascular and inflammatory components.

#### 3.1.2. Glial Activation and Cytokine Dysregulation

Radiation-induced damage to retinal cells and DNA initiates a cascade of inflammatory responses, starting with the activation of resident microglia [19]. These cells, normally responsible for maintaining homeostasis in the retina, undergo a phenotypic transformation in response to ionizing radiation [19]. Activated microglia release an array of pro-inflammatory cytokines, including TNF-α, IL-6, and IL-1β. These cytokines compromise the integrity of the blood-retinal barrier (BRB), which is critical for maintaining the immune-privileged status of the retina [19]. The breakdown of the BRB results in increased vascular permeability, leading to macular edema and further damage to retinal neurons. In addition to cytokine release, key molecular cascades implicated in RM include the activation of the NF-κB signaling pathway and JAK/STAT3 axis, which mediate chronic inflammation and microglial proliferation. Oxidative stress pathways involving NADPH oxidase also contribute to microglial activation and BRB breakdown [19]. Moreover, dysregulation of the CX3CR1/CX3CL1 signaling pathway, a critical regulator of microglial activity, exacerbates these effects [19]. Under normal conditions, this signaling pathway maintains microglial homeostasis, but its disruption in RM amplifies microglial activation and the associated inflammatory responses. Moreover, the neuroinflammatory response appeared quickly after irradiation and seemed to be amplified with time up to 6 months after UM treatment [15]. Indeed, microglial migration in the retina seems to start simultaneously with Müller cell activation [15]. These changes contribute to a chronic inflammatory state that accelerates retinal degeneration and vision loss [19]. In this complex inflammatory network, additional molecular players and mechanisms have emerged as crucial contributors. The mitogen-activated protein kinase (MAPK) cascade, particularly involving p38 and ERK1/2, further promotes the transcription of inflammatory mediators and enhances microglial migration and proliferation [19]. Ionizing radiation also induces mitochondrial dysfunction and activates NADPH oxidases (NOX), leading to the excessive production of reactive oxygen species (ROS). These oxidative stress molecules not only damage retinal cells but also act as intracellular messengers that amplify NF-κB and MAPK signaling, thereby perpetuating inflammation [19]. In parallel, Müller glial cells—key macroglia in the retina—undergo reactive gliosis, releasing additional inflammatory mediators and VEGF, which together foster both neuroinflammation and vascular leakage. This establishes a deleterious feed-forward loop between glial activation, oxidative stress, and BRB disruption [19].

These findings are further supported by recent literature, which demonstrates early inflammatory alterations visible on OCT and OCTA prior to any overt clinical signs of RM, supporting the redefinition of RM as an early neuroinflammatory rather than purely vascular disorder [42]. This evolving perspective underscores the need for early detection and targeted therapeutic strategies addressing inflammation before the development of irreversible structural and functional damage. Recently, several authors have demonstrated that starting anti-VEGF treatment immediately after radiation therapy in high-risk patients may help mitigate visual decline [42,43]. This proactive approach supports the hypothesis that early intervention may alter the natural course of RM. However, it primarily targets the vascular component of RM pathogenesis, leaving underlying neuroinflammatory mechanisms unaddressed. These findings support the idea that while early anti-VEGF therapy may modify the disease course, it does so by acting on only one aspect of a multifactorial process.

### 3.2. Imaging Parameters of Retinal Neuroinflammation in Radiation Maculopathy

#### 3.2.1. Hyperreflective Retinal Foci

**Figure 2:**
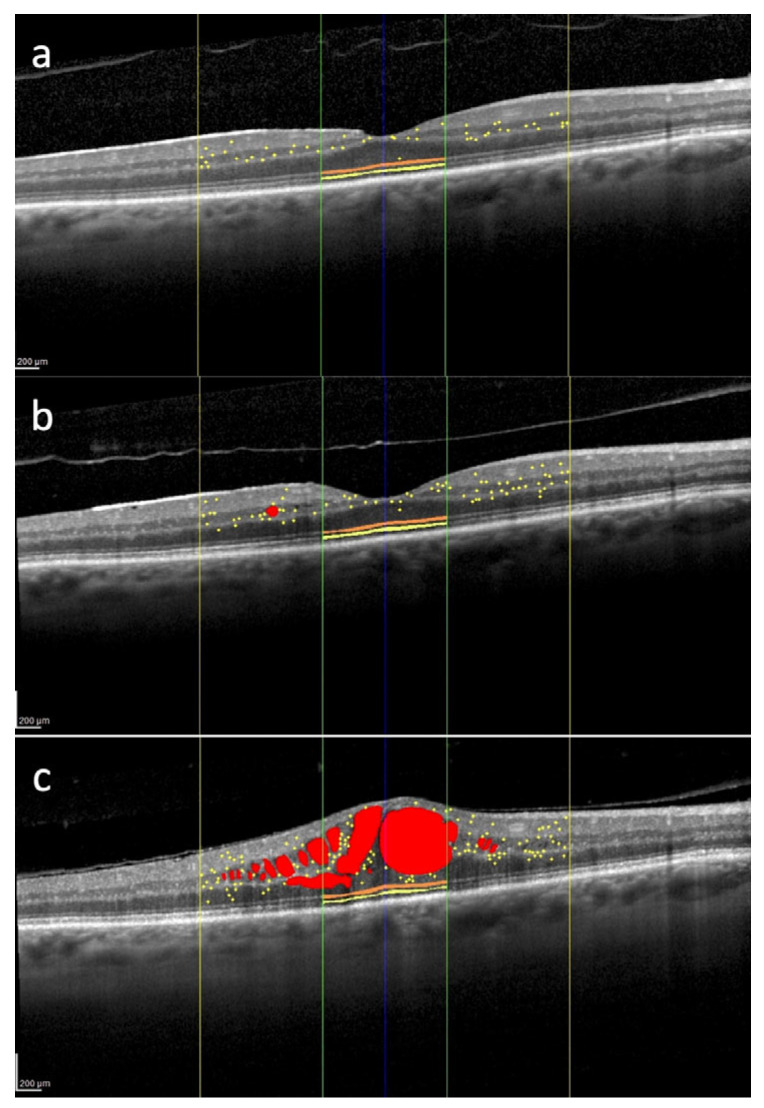
Overview of several spectral domain optical coherence tomography biomarkers evaluated in the macula of the right eye of a 62-year-old patient treated for uveal melanoma. The tumor was located in the inferior quadrant and classified as medium-sized. The patient underwent Iodine-125 plaque brachytherapy, with a total dose of 85 Gy delivered to the tumor apex. Intraretinal fluid (red); hyperreflective retinal foci (yellow dots) localized within the central 3 mm (yellow lines); external limiting membrane (orange) and ellipsoid zone (yellow) localized within the central 1 mm (green lines). 12 months after brachytherapy, hyperreflective retinal foci are 35, and they are mainly located in the inner retina (**a**). 24 months after treatment, there is an increase in the number of hyperreflective retinal foci with their progressive migration toward the outer retina (**b**). 30 months after brachytherapy, there is a further increase in the number of hyperreflective retinal foci with the appearance of center-involved macular edema (**c**). No treatment was administered between timepoints (**b**,**c**). Scale bar: 200 µm.

Hyperreflective retinal foci of inflammatory origin are increasingly recognized as valuable imaging biomarkers for the early detection of retinal neuroinflammation in radiation maculopathy and other macular disorders, including diabetic macular edema [20]. These foci typically appear as isolated, small-sized (<30 µm), moderately reflective spots—comparable to the reflectivity of the retinal nerve fiber layer (RNFL)—and are initially localized in the inner retinal layers. Importantly, they do not correlate with any identifiable anatomical structures on color fundus photography or en face OCT, such as blood vessels or lipid exudates. Current evidence suggests that these HRFs likely correspond to clusters of activated and proliferating microglial cells, thereby providing an in vivo indicator of localized retinal inflammation: (neuro)inflammatory HRF (I-HRF) [43]. Importantly, the number and spatial distribution of I-HRF tend to increase even before the clinical onset of macular edema, providing an opportunity for early detection of disease, as represented in Figure 2. The identification of I-HRF is not only helpful in diagnosing RM at a preclinical stage but also offers insights into the underlying inflammatory processes [16]. By tracking changes in I-HRF number over time, clinicians can monitor disease progression and potentially evaluate the efficacy of interventions aimed at reducing inflammation [16].

#### 3.2.2. Subretinal Fluid

**Figure 3:**
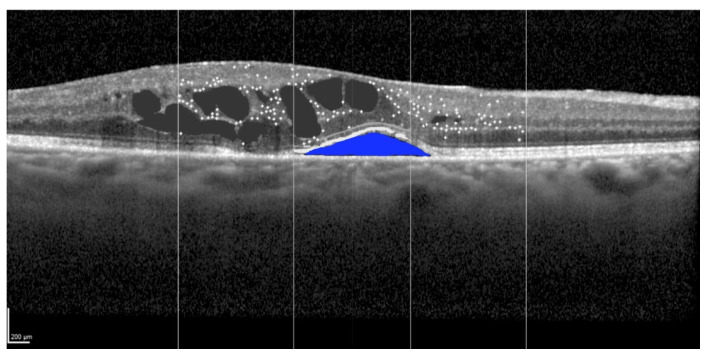
Identification of the subretinal fluid (blue) at spectral domain optical coherence tomography in the left eye of a 58-year-old patient treated for a large-sized choroidal melanoma located in the superior-nasal quadrant. The patient underwent Iodine-125 plaque brachytherapy with a total apex dose of 85 Gy. The subject had previously undergone a cycle of intravitreal anti-VEGF injections, which yielded no anatomical or functional improvement. Scale bar: 200 µm.

Subretinal fluid accumulation is another key feature of advanced RM, reflecting severe disruption in retinal homeostasis [22]. This fluid collects between the neurosensory retina and the retinal pigment epithelium (RPE) due to increased permeability, as represented in Figure 3. Subretinal fluid can exacerbate retinal detachment and photoreceptor damage, further impairing visual function [44]. OCT imaging provides detailed visualization of subretinal fluid, enabling accurate quantification and tracking over time. Persistent subretinal fluid is often associated with more severe stages of RM, signaling a progressive breakdown of the blood–retinal barriers and chronic inflammation [8]. Clinicians can use subretinal fluid characteristics, such as volume and persistence, as markers of disease severity. Additionally, changes in subretinal fluid volume during treatment can indicate therapeutic responses, helping to tailor management strategies effectively. Early detection and intervention aimed at controlling subretinal fluid can mitigate structural damage and improve visual outcomes.

#### 3.2.3. Ischemia

Ischemia plays a central role in the progression of RM, resulting from radiation-induced microvascular damage and capillary dropout [1,15]. Hypoxia caused by ischemia exacerbates retinal neuroinflammation, creating a feedback loop that accelerates retinal degeneration. OCTA is particularly useful for detecting ischemic changes, including capillary non-perfusion, vascular remodeling, and areas of reduced perfusion both in the retina and choroid [29,38].

**Figure 4:**
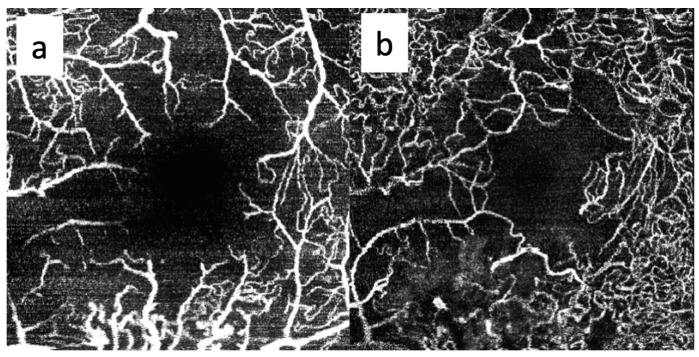
OCTA imaging of the superficial (**a**) and deep (**b**) capillary plexuses in the right eye of a 65-year-old patient treated for a large-sized choroidal melanoma located in the inferotemporal quadrant. The patient underwent Iodine-125 plaque brachytherapy with a total apex dose of 85 Gy. Both plexuses show capillary dropout and enlargement of the foveal avascular zone 20 months after treatment. No intravitreal treatment was administered prior to image acquisition. The patient had no history of diabetes mellitus.

In detail, OCTA offers detailed depth-resolved imaging, enabling the identification of ischemic regions in both superficial and deep retinal vascular plexuses, as stated in Figure 4. These ischemic areas often correspond to regions of retinal thinning and structural abnormalities seen on OCT [31,32,40]. By combining structural and functional imaging, OCTA provides a comprehensive view of the impact of ischemia on retinal health. Therefore, ischemic progression can be monitored longitudinally, and the effectiveness of therapeutic strategies aimed at restoring vascular integrity or reducing hypoxic stress can be evaluated [23,44,45].

Moreover, OCTA is fundamental to assessing the involvement of the choroidal vasculature in RM [45]. Shields et al. reported reduced vessel density in the choriocapillaris at the tumor margins in patients with and without clinically evident RM [32]. In a study including 112 irradiated eyes without visible signs of RM, OCTA analysis of the choriocapillaris revealed areas of signal void in 88% of cases, rarefaction in 94%, and vessel dilation in 41% [40]. These findings suggest that OCTA-detected capillary ischemia may serve as an early marker, preceding the clinical manifestations of RM. Although not an imaging technique, electroretinography (ERG), particularly multifocal ERG (mfERG), provides a valuable functional evaluation of retinal integrity in radiation maculopathy. Reduced amplitudes in both scotopic and photopic responses reflect diffuse inner retinal dysfunction due to radiation-induced ischemia. As demonstrated by Gong et al., early functional changes can be detected by mfERG even in the absence of overt structural damage on OCT, supporting its role as a complementary tool to imaging, especially in preclinical or atypical presentations of RM [25].

## 4. Conclusions

RM is a complex condition that arises as a delayed consequence of ocular irradiation, particularly in the management of UM. While traditionally considered a vascular disorder characterized by capillary occlusion, ischemia, and edema, recent advances have highlighted the crucial role of retinal neuroinflammatory mechanisms in its pathogenesis [1,15,16]. The activation of retinal microglia, the release of pro-inflammatory cytokines, and the breakdown of the BRB contribute to a self-perpetuating cycle of inflammation, neurodegeneration, and vascular instability [16,19].

Multimodal non-invasive imaging techniques, such as OCT and OCTA, have emerged as essential tools for early detection and monitoring of RM [3]. Imaging biomarkers such as I-HRF and subretinal fluid provide valuable insights into the inflammatory and degenerative changes occurring at a subclinical stage, allowing for earlier intervention [12,44]. Additionally, ischemic alterations detectable via OCTA offer a crucial perspective on the progressive vascular compromise affecting both the retina and choroid, further supporting the link between ischemia and neuroinflammation [23,32,41,45,46,47].

Understanding RM as a neurovascular rather than solely a vascular pathology underscores the need for targeted therapeutic approaches that address both vascular and inflammatory components [33,48]. Future research should focus on developing strategies to modulate microglial activation, reduce cytokine-driven inflammation, and preserve neurovascular integrity. The integration of artificial intelligence-assisted imaging analysis holds promise for refining early diagnostic accuracy and optimizing treatment monitoring.

By shifting the paradigm of RM from a purely ischemic model to one incorporating neuroinflammation and neurodegeneration, clinicians can improve patient outcomes by implementing earlier, more effective interventions. Continued advancements in imaging and therapeutic strategies will be critical in mitigating disease progression and preserving visual function in patients affected by radiation-induced retinal damage.

In conclusion, the added value of the present review lies in its integrated perspective on RM, emphasizing the role of early neuroinflammation and its non-invasive retinal imaging correlates. By systematically describing spectral-domain OCT and OCTA biomarkers—such as I-HRF and foveal avascular zone enlargement—this work highlights subclinical disease stages and potential early endpoints for intervention. The review also bridges experimental evidence on glial activation and cytokine dysregulation with real-world imaging findings, promoting a revised model of RM as a progressive neuroinflammatory rather than purely ischemic complication. These insights may guide future diagnostic algorithms and tailored anti-inflammatory strategies in patients undergoing ocular radiation.

## Data Availability

No new data were created in this study. Data sharing is not applicable to this article.

## Abbreviations

RM: radiation maculopathy; UM: uveal melanoma; OCT: optical coherence tomography; OCTA: optical coherence tomography angiography; I-HRF: inflammatory hyperreflective retinal foci; BRB: blood–retinal barrier; RPE: retinal pigment epithelium; RNFL: retinal nerve fiber layer; SASP: senescence-associated secretory phenotype; CX3CR1: CX3C chemokine receptor 1; CX3CL1: CX3C chemokine ligand 1; TNF-α: tumor necrosis factor alpha; IL-6: interleukin-6; IL-1β: interleukin-1 beta; AI: artificial intelligence; DNA: deoxyribonucleic acid; NF-κB: nuclear factor kappa-light-chain-enhancer of activated B cells; JAK/STAT3: Janus kinase/signal transducer and activator of transcription 3; MAPK: mitogen-activated protein kinase; ERK1/2: extracellular signal-regulated kinases 1 and 2; ROS: reactive oxygen species; NOX: NADPH oxidase; VEGF: vascular endothelial growth factor.
